# Supplementary material for: Synthesis and preclinical evaluation of [11C]MTP38 as a novel PET ligand for phosphodiesterase 7 in the brain
Source: Eur J Nucl Med Mol Imaging. 2021 Mar 5;48(10):3101–12. doi: 10.1007/s00259-021-05269-4 (PMC8426238; doi:10.1007/s00259-021-05269-4)
Supplement: Supplementary file 1 — (DOCX 97 kb) [file 259_2021_5269_MOESM1_ESM.docx]

**Supplementary Fig. 1.** Logan’s graphical plot in the monkey brain

Representative (monkey #1) Logan’s graphical plots in the striatum (**a**) and cerebellum(**b**).


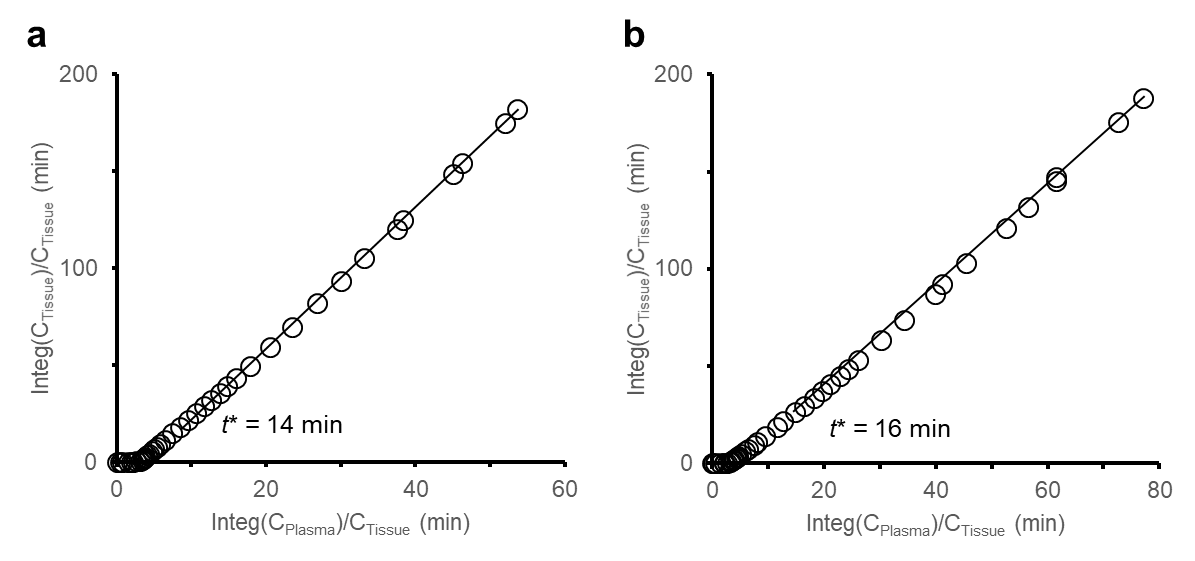


**Supplementary Fig. 2.** Time stability of *V*_T_ estimated by Logan’s plot in the monkey brain

*V*_T_ values in the striatum (circles) and cerebellum (squares) were estimated by Logan’s graphical analysis of dynamic PET data and arterial input function truncated from 90 min to 30 min after radioligand injection. Data are means of two animals. Dotted lines indicate 95% and 100% of the *V*_T_ value estimated with 90-min data.


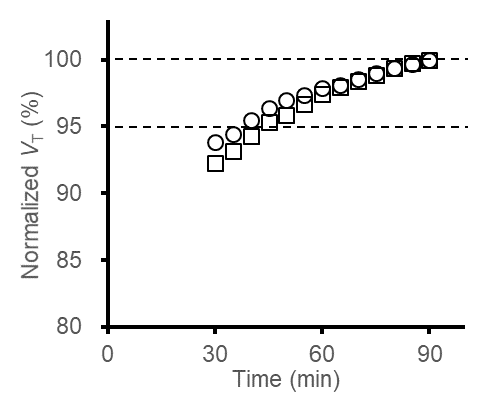


**Supplementary Fig. 3.** Correlations between *BP*_ND_ values calculated by MRTMo and Logan’s plot

Each symbol represents an individual animal. Dotted line indicates the line of identity.


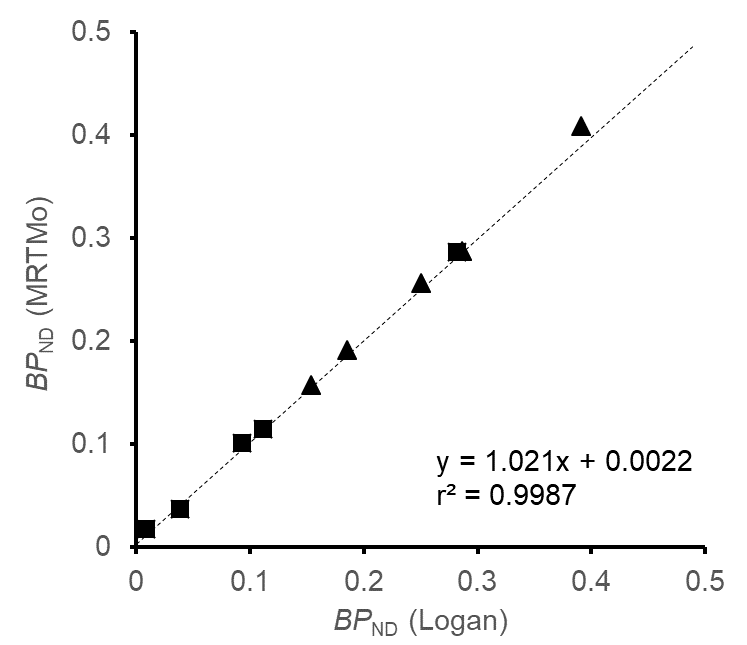


**Supplementary Table 1.** Inhibitory effects of MTP38 on various enzymes, ion channels, receptors, and transporters

| Enzymes, ion channels, receptors, and transporters | Sources | Substrates, ligands | Inhibition (%) at 1 μM |
| --- | --- | --- | --- |
| Adenosine A_1_ | Human recombinant | [^3^H]DPCPX | -12 |
| Adenosine A_2A_ | Human recombinant | [^3^H]CGS-21680 | 4 |
| Adenosine A_3_ | Human recombinant | [^125^I]AB-MECA | 10 |
| Adrenergic α_1A_ | Rat submaxillary gland | [^3^H]Prazosin | 8 |
| Adrenergic α_1B_ | Rat liver | [^3^H]Prazosin | -3 |
| Adrenergic α_1D_ | Human recombinant | [^3^H]Prazosin | -3 |
| Adrenergic α_2A_ | Human recombinant | [^3^H]Rauwolscine | 4 |
| Adrenergic β_1_ | Human recombinant | [^125^I]Cyanopindolol | -2 |
| Adrenergic β_2_ | Human recombinant | [^3^H]CGP-12177 | -14 |
| Androgen (Testosterone) | Human LNCaP clone | [^3^H]Methyltrienolone | 1 |
| Bradykinin B_1_ | Human IMR-90 cells | [^3^H](Des-Arg^10^)-Kallidin | 9 |
| Bradykinin B_2_ | Human recombinant | [^3^H]Bradykinin | 2 |
| Calcium Channel L-Type,  Benzothiazepine | Rat brain | [^3^H]Diltiazem | 16 |
| Calcium Channel L-Type,  Dihydropyridine | Rat cerebral cortex | [^3^H]Nitrendipine | 7 |
| Calcium Channel N-Type | Rat frontal brain | [^125^I]ω-Conotoxin GVIA | 1 |
| Cannabinoid CB_1_ | Human recombinant | [^3^H]SR141716A | -6 |
| Dopamine D_1_ | Human recombinant | [^3^H]SCH-23390 | -14 |
| Dopamine D_2S_ | Human recombinant | [^3^H]Spiperone | 4 |
| Dopamine D_3_ | Human recombinant | [^3^H]Spiperone | 0 |
| Dopamine D_4.2_ | Human recombinant | [^3^H]Spiperone | -3 |
| Endothelin ET_A_ | Human recombinant | [^125^I]Endothelin-1 | -10 |
| Endothelin ET_B_ | Human recombinant | [^125^I]Endothelin-1 | 3 |
| Epidermal Growth Factor (EGF) | Human A431 cells | [^125^I]EGF | 1 |
| Estrogen ERα | Human recombinant | [^3^H]Estradiol | -1 |
| GABA_A_, Flunitrazepam, Central | Rat brain  (minus cerebellum) | [^3^H]Flunitrazepam | -2 |
| GABA_A_, Muscimol, Central | Rat brain  (minus cerebellum) | [^3^H]Muscimol | -10 |
| GABA_B1A_ | Human recombinant | [^3^H]CGP-54626 | -10 |
| Glucocorticoid | Human recombinant | [^3^H]Dexamethasone | 0 |
| Glutamate, Kainate | Rat brain cortex | [^3^H]Kainic acid | -2 |
| Glutamate, NMDA, Agonism | Rat cerebral cortex | [^3^H]CGP-39653 | -3 |
| Glutamate, NMDA, Glycine | Rat cerebral cortex | [^3^H]MDL 105,519 | -15 |
| Glutamate, NMDA, Phencyclidine | Rat cerebral cortex | [^3^H]TCP | -5 |
| Histamine H_1_ | Human recombinant | [^3^H]Pyrilamine | -20 |
| Histamine H_2_ | Human recombinant | [^125^I]Aminopotentidine | -9 |
| Histamine H_3_ | Human recombinant | [^3^H]N-α-Methylhistamine | 9 |
| Imidazoline I_2_, Central | Rat cerebral cortex | [^3^H]Idazoxan | -8 |
| Interleukin IL-1 R1 | Human recombinant | [^125^I]Interleukin-1β | -5 |
| Leukotriene, Cysteinyl CysLT_1_ | Human recombinant | [^3^H]LTD_4_ | 4 |
| Melatonin MT_1_ | Human recombinant | [^125^I]2-Iodomelatonin | 7 |
| Muscarinic M_1_ | Human recombinant | [^3^H]N-Methylscopolamine | -20 |
| Muscarinic M_2_ | Human recombinant | [^3^H]N-Methylscopolamine | -9 |
| Muscarinic M_3_ | Human recombinant | [^3^H]N-Methylscopolamine | -21 |
| Neuropeptide Y Y_1_ | Human SK-N-MC cells | [^125^I]Peptide YY | -13 |
| Neuropeptide Y Y_2_ | Human KAN-TS cells | [^125^I]Peptide YY | 6 |
| Nicotinic Acetylcholine | Human IMR-32 cells | [^125^I]Epibatidine | -8 |
| Nicotinic Acetylcholine α1, Bungarotoxin | Human RD cells | [^125^I]α-Bungarotoxin | 15 |
| Opiate δ_1_ (OP1, DOP) | Human recombinant | [^3^H]Naltrindole | -4 |
| Opiate κ (OP2, KOP) | Human recombinant | [^3^H]Diprenorphine | 9 |
| Opiate μ (OP3, MOP) | Human recombinant | [^3^H]Diprenorphine | 4 |
| Phorbol Ester | Mouse brain | [^3^H]PDBu | -7 |
| Platelet Activating Factor (PAF) | Human platelets | [^3^H]PAF | 4 |
| Potassium Channel [K_ATP_] | Hamster pancreatic  HIT-T15 beta cells | [^3^H]Glyburide | 5 |
| Potassium Channel hERG | Human recombinant | [^3^H]Astemizole | 17 |
| Prostanoid EP_4_ | Human recombinant | [^3^H]Prostaglandin E_2_ (PGE_2_) | 8 |
| Purinergic P2X | Rabbit urinary bladder | [^3^H]α, β-Methylene-ATP | -3 |
| Purinergic P2Y | Rat brain | [^35^S]ATP-αS | -20 |
| PDE4 | Rat brain | [^3^H]Rolipram | 74 |
| Serotonin (5-Hydroxytryptamine) 5-HT_1A_ | Human recombinant | [^3^H]8-OH-DPAT | -5 |
| Serotonin (5-Hydroxytryptamine) 5-HT_2B_ | Human recombinant | [^3^H]Lysergic acid diethylamide (LSD) | -1 |
| Serotonin (5-Hydroxytryptamine) 5-HT_3_ | Human recombinant | [^3^H]GR-65630 | -11 |
| Sigma σ_1_ | Human Jurkat cells | [^3^H]Haloperidol | 6 |
| Sodium Channel, Site 2 | Rat brain | [^3^H]Batrachotoxinin | 1 |
| Tachykinin NK_1_ | Human recombinant | [^3^H]Substance P | 9 |
| Thyroid Hormone | Rat liver | [^125^I]Triiodothyronine | 7 |
| Transporter, Dopamine (DAT) | Human recombinant | [^125^I]RTI-55 | 22 |
| Transporter, GABA | Rat cerebral cortex | [^3^H]GABA | -2 |
| Transporter, Norepinephrine (NET) | Human recombinant | [^125^I]RTI-55 | 6 |
| Transporter,  Serotonin (5-Hydroxytryptamine) (SERT) | Human recombinant | [^3^H]Paroxetine | -14 |
